# Cases Cured by Metals

**Published:** 1881-07

**Authors:** Wm. P. Wesselhoeft

**Affiliations:** Boston


### CLINICAL BUREAU.

### CASES CURED BY METALS.

WM. P. WESSELHOEFT, M. D., BOSTON.

CUPRUM MET.—C. F. æt. 65. Robust, healthy man.

*November* 19th, 1879. Was attacked three days ago, very suddenly, with watery running from the nose, asthmatic breathing, spasms of suffocation, cough with gagging and expectoration of tough, tenacious mucus.

The attacks come on very suddenly every hour or two, and are worse nights, waking him suddenly with coughing and gagging.

There are coarse mucus râles throughout both lungs, occasionally sibilant, but characterized mostly by coarse bubblings, which can be heard some distance from the bed.

He is very anxious, and fears he will choke to death in one of these attacks. These paraoxysms of dyspnœa pass off very suddenly after a furious effort of gagging and coughing, followed by great prostration. Then follows an interval of comparative ease, and he gets, perhaps, a little nap between the attacks, but the bubbling respiration is constant all the time.

Has disgust for all food, with some nausea Tongue thickly coated white. No thirst. Pulse 100.

Has been under allopathic treatment, but steadily growing worse.

Had a similar attack, but much less distressing, fifteen years ago, from which he slowly recovered after many months, and during which his life was despaired of.

I gave *Antimonium tartaricum*, c. m. (S.) in water, a teaspoonful to be taken every two hours, at 8 o'clock, P. M.

Next morning, November 20, has had not quite as many coughing and gagging spells, but the dyspnœa and rattling breathing are fully as severe. Complains of terrible sinking faintness in abdomen with constant nausea, and the same aversion to food.

I had studied the case very carefully the evening before, and determined to give *Cuprum* if *Antim. tart.* produced no decided change.

I selected *cuprum* chiefly on account of the paraoxysmal character of the attacks, coming suddenly and decreasing suddenly; the convulsive spasmodic gagging, and the coarse mucus râles.

This metal was given in a single dose, c. m. (S.) potency. It proved to be the simillimum. He had not a single spasm afterward, slept the night through, and on the 26th of November not a vestige of rattling remained in his lungs, and he went his way rejoicing.

CUPRUM MET.—*C. A. F.*, aged six weeks, a grandson of the above patient.

*February* 16th, 1880. When two weeks' old, was attacked with spasmodic cough and dry, stuffed nose. The latter symptom was so distressing, that the child had the greatest difficulty in nursing. Indeed, the mother's milk was mostly given him by the spoon, after being pumped from the breast. The child was thought to have the whooping-cough, and had been treated by a homœopathist without material change; the cough and obstructed nose remaining the same. As the mother was suffering greatly with pain in her breast and nipples, owing to the frequent and unnatural process of obtaining nourishment for the child, my first thought was to relieve this obstinate nose trouble by *Sambucus*. After using this remedy for thirty-six hours (in solution, every three hours) there was not the slightest change.

During this time, however, I had opportunity of observing several paraoxysms of coughing. The attack was preceded by short, whistling breathing, followed by violent paroxysms of coughing, the face becoming a deep red color, the child's body rigidly stretched out. All this reminded me of *Cuprum*, remembering at the same time the success of the remedy with the grandfather three or four months before. I gave the remedy in the same potency, in water, every three hours a teaspoonful during twenty-four hours. The effect was quite as gratifying and astonishing as before. The child was discharged cured, on the 22d of February, and could take the breast without difficulty.

On the 1st of April, 1881, a little more than year after this attack, he had another similar, but lighter one. The same remedy relieved in the same potency.

Hahnemann mentions "stuffed nose" as one of the prominent symptoms cured by this remedy, in his introduction to *Cuprum* ("chronic diseases"). It may become a valuable remedy in the very common trouble of infants, usually called "sniffles," when other remedies fail. I have not yet had occasion to test it.

Although both of these cases were of an acute nature, one of them having lasted only three days, the other four weeks, yet I cannot well conceive how its direct curative effect of the metal in both cases can be denied, unless remedial action is declared untrue in any potency above the 6th trituration, because it cannot be seen under the microscope. I, for one, prefer the test in disease. In my humble opinion, it was *Cuprum met.* alone, potentized from the 3d trituration which effected the cures, because it was the *most homœopathic* remedy to both cases. The preparation used also convinced me that the fluxion potencies are as reliable as those prepared by succussion.

PLUMBUM METALLICUM: J. J. C., boy, aged 11. Light brunette. The past three years, has had attacks of severe pain in abdomen, coming in paroxysms which last several weeks. Is sometimes entirely free from the pain for a week or ten days at a time. The pain is in the region of the navel, of a sharp cutting or contracting character, with a sensation of drawing inward towards the spine.

Is worse from any excitement, and when attending school; is diffident, and afraid of the dark. Has a tolerable appetite, but afraid to eat, fearing the pain will be increased by it, which his mother, however, does not think is the case. Is generally worse mornings. Is subject to harsh, dry cough, and takes cold easily. All functions normal.

*September* 24, 1878. I gave him a dose of *Plumbum met.*, c. m. (S.), selected especially on account of the contracting pains in the region of the navel.

On the 5th of October, reports pain much improved. Has a good appetite, and less afraid to eat.

Entire freedom from pain continued, under *sac. lact.*, till the 7th of of January, 1879, when, after eating freely of candy, the pain returned, and he received another dose of *Plumbum met.*, c. m., which relieved him until the 28th of January, so that he was practically without pain from September 24, 1878, till January 28, 1879—four months.

After this, the pain returned at irregular intervals less severe, but accompanied by a craving hunger. *Plumbum* given again did not relieve him. It was followed by *Psorinum*, and later *Sulphur*, with indifferent success. The cure, however, was interrupted by the patient's leaving in March, 1879, for Europe, since which time I have heard nothing from him.

Although this case cannot be enumerated among the cures by *Plumbum met.*, it had, nevertheless, a most marked effect, and both patient and mother were gratified with the result.

I merely record it as a *case relieved* by a highly potentized metal, and which I had every hope of curing, had time and opportunity allowed a further selection of antipsorics.

PLATINUM: Mrs. R. D. R., æt. 25. Brunette, healthy color, good complexion, and well nourished.

*May 4th,* 1880. Has been afflicted with obstinate constipation of bowels from her youth. Has purged more or less all her life. Under homœopathic treatment for three months, before applying to me, without relief.

Is five or six days without a desire to go to stool. No power in rectum to expel the stool. Feels as if the load of feces was lying at the opening of the anus, unable to expel it unless she has taken an aperient medicine, or used an enema. The stool is of large form, composed of small and very hard balls.

Since her confinement, three months ago, feels very weak. During her pregnancy, took aperient medicine every day or two. Has little appetite, and nauseated after eating. Feels particularly miserable about 11 A. M., with a hungry, faint feeling, but cannot eat. This feeling lasts till past noon.

Had ulcers on both legs between her eleventh and twelfth year. Has occasional sharp, stitching pains in left ovarian region. Menstruation is normal.

Knowing that she must have had *Nux vom.* "*ad nauseam*" from her previous physician, that remedy was excluded without further thought. The faint feeling at noon decided me to give her one dose of *Sulphur,* c. m. to be dissolved in four table-spoonfuls of water, a table-spoonful to be taken morning and evening until used up, followed by *Sac. Lac.* for one week, with directions to take no other medicine, nor use injections. Her diet, which was correct, was not changed.

*May 12th.* No improvement. After five days had a very difficult stool; anus is sore, and several small piles have appeared on edge of anus. Tired ache in upper sacral region. Gave *Sac. lact.*

*May 21st.* Had no stool until four days ago; could not expel it, until she took an enema of tepid water. The following day had a stool without enema, which she thought was part of the feces remaining from the incomplete stool of the day previous. *White glairy mucus from vagina* followed the last stool. *Pain in sacrum,* and soreness of anus continues, *the former worse in early morning.* Faintness at noon is better. Gave *Natrum mur.,* c. m. (S.), one dose dry, followed by *Sac. lact.*

*June 2d.* Has had five natural stools without enemas, occurring every other day. Decidedly more power to expel stool. No soreness of anus, or piles. This improvement continued under *sac. lact.* till

*June 28th.* Reports stools occur only once in three days, *larger*

*in form*, and of lighter color; more effort required to expel stool. *Wakes between three and five A. M.* with nausea. Gave *Kali-carb.* c. m. (S.), one dose dry. This remedy again relieved her, and she had easy stools every forty-eight hours. During the menses, bowels moved daily.

*August 22d.* Reports by letter from the West, where she has been traveling for several weeks, that her bowels are again very constipated, having an operation but once in five to seven days, with the same difficulty of expulsion. No return of pain in sacrum or soreness of anus.

Dr. J. B. Bell, in my absence, sent her one dose of *Platinum* c. m. (S.) followed by *Sac. lact.* On the

*Fourth of October*, reports great improvement after *Platinum.* Has now *every day* a natural stool; and has had since receiving the last medicine. Stools are normal in size and require but little effort at ejection.

I saw this lady a month ago, when she came to engage me for her confinement, being at that time over two months pregnant. She told me her bowels had been in excellent order up to the second week of pregnancy, since which time they had moved comfortably every other day. With this state of things she was perfectly satisfied, particularly when she called to mind the torture and wretched condition during her previous pregnancy.

This case, greatly relieved by *Natrum mur.*, followed by *Kali-carb.*, was undoubtedly cured by *Platinum*; at all events the cure was completed by this metal, although given only on the indication, "constipation worse while traveling." It not only relieved the symptom *while* traveling, but so regulated this function, that for the first time since early childhood she had daily stools without pain or inconvenience.

In the face of such facts, may we not be allowed to differ from those, who not only slur Hahnemann's greatest discovery, the dynamization of drugs, but would make us believe that the remedial action of metals *must* end with the sixth centesimal trituration, because the substance cannot be seen under the field of the microscope after this division!

The controversy, so long and bitterly waged regarding the dose, appears at last to have some show of coming to an end. If the astonishing results of Prof. Jæger's observations with the chronoscope can be confirmed by other observers, we shall hear nothing more of chemical or spectral analysis, much less of microscopic researches to discover the last poor atom in a homœpathic potency.

Jæger's results have all been obtained by *olfaction*, and the machine has recorded the nerve oscillations with such wonderful accuracy, unison and agreement, that there is every reason to hope not only the potentized substance itself may be discerned by its own characteristic cures, but the degree of potency employed in each experiment.

We may soon hope for a series of experiments by Dr. Fellger of Philadelphia, and Dr. Siemsen of Copenhagen, who have both received instruments through Prof. Jæger.

To have Hahnemann's observations confirmed, for which he has been so generally maligned, that olfaction is often the better mode of administering the remedy, will be of incalculable value for an additional estimate of this great man's genius in observing the most delicate phenomena of disease.

Prof. Jæger, himself an unwilling convert to the powers of homœopathic attenuations, makes the following remarks regarding his persecutions and the malignity with which his discoveries have been received. He, like Hahnemann, challenges his opponents to make the experiment, but to make it as he directs. These are his words:

"I do not expect or wish that my assertions should be blindly accepted; but I think I have a right to demand that these assertions should be examined, and proved or disproved. I have a right to assert that he who judges without having experimented, does not deserve the name of scientist—not even that of a man of honor. I well know how inconvenient and uncomfortable it will be to many to have my investigations confirmed; this, however, gives no man the right to heap upon me personal abuse. Honorable men must endure the truth, no matter how bitter it may prove.

"When Harvey discovered the circulation of the blood; Sœmmering invented the telegraph; Pleysonell proclaimed the animal nature of the Corall; when Robert Mayer found the equivalent of heat, etc.; these discoveries were not only received with malicious phrases, but the discoveries pronounced consummate nonsense. The names of the discoverers were held up to public scorn by attaching to them insulting attribute. Harvey was called "the circulator" for several centuries; attempts were made to dispossess him of office and honors, to alienate his dependants, and had not the King interferred in his behalf, he would have died a ruined man.

"If any one believes that these dark ages have passed away; that such things are impossible in our enlightened century, I regret to be obliged to say that affairs are precisely the same now as they were then."

This sounds very much as if poor Jæger was undergoing similar persecutions with which Hahnemann was honored in Germany. Hahnemann found an asylum in France; Prof. Jæger may be obliged to seek one in America. If what he proclaims proves true, we homœopaths of America should pension him liberally during his lifetime.

Should, however, the entire work and observations of Jæger be disproved by a "consensus of the competent," no Hahnemannian will wince, for he has the *certain knowledge* that metals do act in the highest potencies, and that we have not yet found the limit of their divisibility.

